# Network pharmacology analysis and in vitro verification of the anti-sarcopenia effects of formononetin

**DOI:** 10.1186/s40643-025-00965-7

**Published:** 2025-11-03

**Authors:** Yan Fang, Zhu Wei, Zhang Lei

**Affiliations:** 1 Kunshan Rehabilitation Hospital, Kunshan, China; 2 Kunshan Hospital of Traditional Chinese Medicine, Kunshan, Jiangsu China

**Keywords:** Sarcopenia, Formononetin, Network pharmacology, C2C12 cells, Antioxidant, Molecular mechanism

## Abstract

Sarcopenia (SP) associated with functional impairment is highly prevalent; however, therapeutic strategies addressing this condition remain limited. Inflammation and oxidative stress are the key contributors. Suitably, formononetin (FMN) offers diverse benefits, including antioxidant, anti-apoptotic, and anti-inflammatory properties. Therefore, this study used network pharmacology to identify 81 potential target genes for FMN to alleviate SP. Serine/threonine-protein kinase 1 (AKT1), epidermal growth factor receptor (EGFR), and sirtuin 1 (SIRT1) as the core targets. Kyoto Encyclopedia of Genes and Genome analysis indicated that FMN primarily affects SP via the interleukin (IL)-17, PI3K-Akt and FoxO signalling pathways. Cell studies revealed that FMN reduces IL-6 release and boosts superoxide dismutase activity, thereby enhancing C2C12 skeletal muscle cell vitality. FMN intervention also enhanced AKT1 and SIRT1 gene and protein expression, decreased muscle-specific RING finger protein-1 gene expression, and increased EGFR protein expression. This suggests its anti-inflammatory and antioxidant effects in dexamethasone-treated C2C12 cells, potentially preventing muscle atrophy by inhibiting protein breakdown. These findings highlight the promising multi-target role and molecular mechanism of FMN in the treatment of SP and suggest future clinical applications.

**Graphical Abstract:**

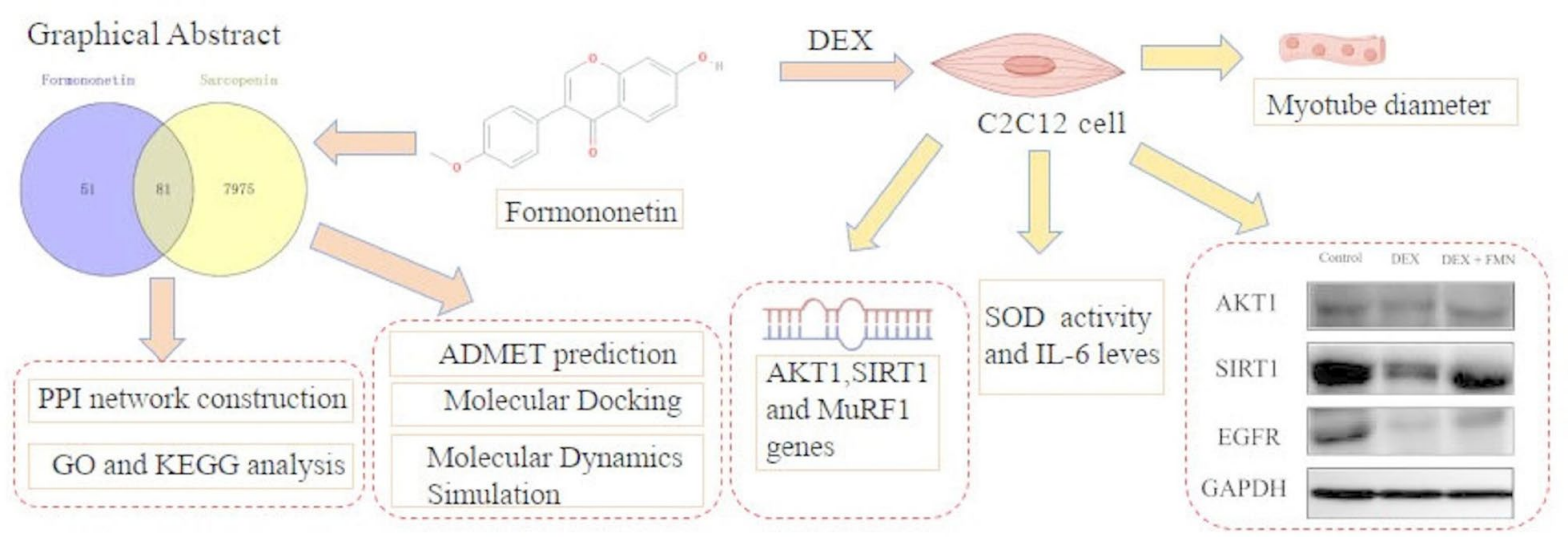

## Introduction

Sarcopenia (SP) is an age-related disease characterised by muscle loss and dysfunction (Muraki 2023), often leading to increased frailty in older adults (Dong et al. 2024). Muscle mass begins to decline around the age of 40 years, with a prevalence of up to 50% in older adults (Kim and Choi 2013; Chung et al. 2025). This condition increases the risk of complications, such as cardiovascular disease, depression, and hypertension (Zeng et al. 2024; Endo et al. 2021). Factors, such as malnutrition, chronic inflammation, oxidative stress, and reduced muscle cell activation, contribute to SP (Chung et al. 2025; Caballero-García et al. 2021). Although no specific SP drugs exist, resistance exercises, protein supplements, and vitamin D are the recommended strategies. Common high doses of testosterone may increase muscle strength; however, this may result in severe side effects, such as prostate disease and thrombosis (Morley 2016). Therefore, further studies are required to develop novel therapeutic agents.

In traditional Chinese medicine, SP is called “weakness syndrome” or “deficiency fatigue”, which refers to muscle nourishment loss due to essence and blood depletion. Chinese medicine has long used herbs to treat “flaccidity” to address age-related issues, such as osteoporosis. These herbs include kidney-nourishing dodder, which contains flavonoids, such as luteolin and quercetin (Yang et al. 2022). The flavonoids in *Epimedium* act as natural antioxidants by resisting free radicals and boosting catalase (CAT) and glutathione peroxidase (GSH-Px) activities (Yang et al. 2020). Formononetin (FMN) is a kidney-nourishing natural isoflavone found in plants, such as red clover (*Trifolium pratense* L.), Astragalali Radix (Lv et al. 2011; Liang et al. 2019), and raspberries (Chen et al. 2024). The compound has antioxidant, antitumour, and anti-inflammatory properties (Zeng et al. 2024; Ong et al. 2019; Endo et al. 2021). FMN offers health benefits through both oestrogen-dependent and-independent mechanisms and has been effective in treating neurological conditions, such as ischaemic stroke and Alzheimer’s disease (Machado Dutra et al. 2021; Ma and Wang 2022; Huang et al. 2018; El-Bakoush and Olajide 2018; Tay et al. 2019; Sun et al. 2012).

Considering the anti-inflammatory and antioxidant properties of FMN, the compound may also be used to treat SP, which involves inflammation, oxidative stress, and muscle cell apoptosis. Although FMN has shown promise in addressing muscle atrophy in chronic kidney disease, additional research is needed to understand its effects on SP due to other causes (Liu et al. 2021). Network pharmacology (NP) uses multitarget biological networks to uncover connections between drugs, diseases, and targets, thereby aiding drug repurposing and identifying new therapeutic targets (Joshi et al. 2025; Chen et al. 2022). This study used human RNA sequencing and NP to identify therapeutic targets and confirmed the effectiveness of MG-132 and troglitazone in treating SP in C2C12 muscle cells (Ceyhan et al. 2024). Similarly, another study employed NP and data mining to explore Chinese medicine for SP treatment owing to the lack of specific drugs (Zhou et al. 2023). Thus, this study aimed to use NP to identify targets for FMN to treat SP, explore drug-target-pathway relationships, and validate key targets through in vitro experiments to understand the mechanisms of FMN and support drug development.

## Materials and methods

### Drug and disease target screening

The Simplified Molecular Input Line Entry System (SMILES) for FMN was sourced from PubChem (https://pubchem.ncbi.nlm.nih.gov, accessed on 19 January 2025), and potential targets were predicted via Traditional Chinese Medicine Systems Pharmacology (TCMSP, https://old.tcmsp-e.com/tcmsp.ph, accessed on 19 January 2025) and SwissTargetPrediction (STP, http://www.swisstargetprediction.ch/, accessed on 19 January 2025) and were standardized in UniProt (https://www.uniprot.org, accessed on 21 January 2025) (Li et al. 2022). The keyword “Sarcopenia” was used to find relevant targets in the Comparative Toxicogenomics Database (CTD, https://ctdbase.org/, accessed on 23 January 2025), Online Mendelian Inheritance in Man (OMIM, https://www.omim.org/, accessed on 23 January 2025), and GeneGards (https://www.genecards.org/, accessed on 23 January 2025) databases, with duplicates removed to identify the final disease targets (Wang et al. 2025).

### Acquisition of cross-targets and construction of action network

The obtained drug and disease targets were input into Venny 2.1.0 (https://bioinfogp.cnb.csic.es/tools/venny/, accessed on 25 January 2025) to find common targets and create a Venn diagram. These common targets were then visualized in Cytoscape3.7.2 to construct a drug-target-disease network diagram (Qian et al. 2022).

### Establishment and analysis of protein–protein interaction (PPI) networks

To identify core goals and their relationships, PPI analysis was conducted using the STRING 12.0 database (https://cn. string-db. org/, accessed on 25 January 2025) for FMN treatment targets of SP, focusing on *Homo sapiens* with an interaction score > 0.7 and hiding stray targets (Liu et al. 2023). Data were extracted as TSV files, and the PPI network was visualised using Cytoscape 3.7.2. (accessed on 25 January 2025). Key topological parameters were analysed using the CytoNCA plug-in, and the core target gene was identified by examining Degree Centrality (DC), Closeness Centrality (CC), and Between Centrality (BC) (Chang et al. 2025).

### Gene ontology (GO) and kyoto encyclopedia of genes and genomes (KEGG) enrichment analysis

The Database for Annotation, Visualization, and Integrated Discovery (DAVID) 6.8 database (https://davidbioinformations.nih.gov/, accessed on 20 June 2025) was used to elucidate the molecular mechanisms of FMN (Zhang et al. 2024). GO functional enrichment and KEGG pathway analyses were conducted following the integration of differentially expressed genes identified through the intersection of target datasets and the Gene Expression Omnibus (GEO) database (https://www.ncbi.nlm.nih.gov/geo/, accessed on 18 June 2025). The results were visualised using the Microinformatics Platform (http://www.bioinformatics.com.cn, accessed on 22 June 2025) (Wang et al. 2024a, b). All GO terms, including biological processes, molecular functions, and cellular components were assessed (Park et al. 2016). Only GO terms and KEGG pathways with P-values < 0.05 were considered for further research (Shameer and Sowdhamini 2012).

### Molecular docking (MD)

MD is a useful method for predicting the binding affinity of small molecule ligands for receptor sites (Guedes et al. 2014; Xue et al. 2022). MD was used to explore the connection between the three core targets and FMN. First, the three-dimensional structure of the human protein was downloaded from the Protein Data Bank (PDB, http://www.rcsb.org/, accessed on 8 February 2025) and the compound structure was downloaded from PubChem. After removing water molecules, preparing protein structures, and minimising energy, compounds were analysed using AutoDockTools1.5.6 (accessed on 10 February 2025). AutoDockVina 1.1.2 (accessed on 10 February 2025) was used to determine receptor-ligand docking, and PyMol 2.3.4 (accessed on 10 February 2025) was used to visualise potential drug-target interactions (Wang et al. 2024a, b).

### Molecular dynamics simulations (MDS)

MDS was conducted using Gromacs 2019.6 (accessed on 16 June 2025) with the AMBER99SB force field and TIP3P water model. Protein and ligand topologies were obtained, solvent water molecules were added, and ions were included to ensure charge neutrality. Energy minimisation was performed on the solvated system at 300 K using the steepest descent method over 50,000 steps, with the LINCS algorithm constraining the system (Liu et al. 2024a, b, c). The simulation was run for 100 ns with a 2-fs step length, storing conformations every 20 ps. Visualisation was performed using Pymol 2.3.4 (accessed on 23 June 2025).

### Prediction of pharmacokinetics and toxicity

Absorption, distribution, metabolism, excretion, and toxicity (ADMET) analyses constitute a critical phase in assessing the pharmacokinetic properties and safety profiles of candidate compounds during drug development. Following the acquisition of SMILES notation for core active ingredients from PubChem, the Swiss ADME database (http://www.swissadme.ch/, accessed on 23 June 2025) (Tshiluka et al. 2025) and the pKCSM database (http://structure.bioc.cam.ac.uk/pkcsm, accessed on 23 June 2025) (Pires et al. 2015) were used to predict the clinical applicability of FMN.

### Materials

The C2C12 mouse myoblast cell line was obtained from QuiCell (Shanghai, China). Foetal bovine serum (FBS) was obtained from Hongquan (Guangzhou, China). Trypsin-ethylenediaminetetraacetic acid digestion solution (0.25%), penicillin/streptomycin (100 ×), dimethyl sulfoxide (DMSO), phosphate-buffered saline (PBS), radioimmunoprecipitation assay (RIPA) lysis solution, and protein-loading buffer (4 ×) were obtained from Solarbio (Beijing, China). Dexamethasone (DEX), interleukin (IL)-6, superoxide dismutase (SOD), glyceraldehyde-3-phosphate dehydrogenase antibody (dilution 1:2500), serine/threonine-protein kinase 1 (AKT1, dilution 1:1000), sirtuin 1 (SIRT1, dilution 1:1000), epidermal growth factor receptor (EGFR, dilution 1:1000), and horse radish peroxidase (HRP)-labelled secondary antibodies (dilution 1:1000) were acquired from Beyotime (Shanghai, China). Cell Counting Kit-8 (CCK-8) was obtained from Signalway Antibody (SAB, Maryland, USA). SYBR Green polymerase chain reaction (PCR) and bicinchoninic acid (BCA) protein quantification kits were obtained from Thermo Fisher Scientific (Waltham, MA, USA), the RT reverse transcription kit was obtained from Fermentas (Thermo Fisher Scientific, Waltham, MA, USA), and TRIzol reagent was obtained from Invitrogen (Thermo Fisher Scientific, Waltham, MA, USA). FMN (purity > 98%) was purchased from Yuanye Biotechnology Co., Ltd. (Shanghai, China).

### Cell culture

Based on existing research methods (Chen et al. 2020), C2C12 myoblasts were grown in Dulbecco’s Modified Eagle’s Medium (DMEM) with 10% FBS at 37 °C and 5% CO_2_ until fully confluent. At 90% confluence, differentiation into myotubes was initiated by switching to DMEM with 2% horse serum for six days, with medium changes every other day.

### Cell viability determination

The cotreatment effect of DEX and FMN was tested by CCK-8 assay according to previously described instructions (Chen et al. 2020; Sun et al. 2017). C2C12 cells (1 × 10^4^ cells/well) were seeded into 96-well platesand incubated overnight. Cells were exposed to different concentrations of FMN (0,25,50,75 and 100 μM) for 24 h or DEX (0, 125, 250, 500, 1000 μM) for 4 h. At the same time, in order to illustrate basic level of cell viability, the control group was treated with DMSO or PBS separately (Long et al. 2025). Following treatments, 10 μL of CCK-8 solution was added and incubated for 90 min. Subsequently, the absorbance was measured at 450 nm to evaluate cell viability.

### Morphological analysis

Cells (1 × 10^5^ per well) were seeded into a 24-well plates and exposed to 75 μM FMN for 24 h. Myotube cells were then treated with 250 μM DEX for 4 h to induce atrophy, both with and without FMN. Muscle fibre images were captured from three randomly selected microscopic areas, and morphological changes were observed and photographed using a 200 × inverted microscope (Tseng et al. 2019).

### Treatment of DEX and FMN

C2C12 myotubes were divided into three groups: a control group incubated in serum-free medium (vehicle-only), a DEX group treated with 250 µM DEX, and a DEX + FMN group treated with 250 µM DEX and 75 µM FMN. All groups were incubated at 37 °C in serum-free medium for 24 h before cell collection (Lee et al. 2018).

### Detection of SOD activity and IL-6 content

Following the SOD assay kit instructions, 20 µL of sample was added to 96-well plates. Thereafter, samples 200 µL of water-soluble tetrazolium working solution and 20 µL of enzyme solution were added. Plates were then incubated at 37 °C for 20 min. SOD activity was measured at 450 nm using an enzyme-labelled instrument (Kim et al. 2023). IL-6 content was measured as per the enzyme-linked immunosorbent assay (ELISA) kit instructions.

### Real-time polymerase chain reaction (PCR) assays

Cells were collected, and RNA was extracted using TRIzol reagent. Subsequently, complementary DNA (cDNA) was synthesised from the RNA using reverse transcriptase. Real-time PCR amplification was performed using the SYBR Green chimeric fluorescence method. Briefly, the reaction mixture consisted of 11 μL of double-distilled H_2_O, 10 μL of SYBR Green Mix, 1 μL of the forward primer, 1 μL of the reverse primer, and 2 μL of template cDNA, resulting in a total volume of 25 μL. The reaction conditions were as follows: initial denaturation at 95 °C for 1 min, followed by 40 cycles of denaturation at 95 °C for 15 s and annealing at 60 °C for 15 s (Okpako et al. 2024). The primer sequences are listed in Table 1. Relative gene expression levels were calculated using the 2^−ΔΔCT^ method, with each sample analysed in triplicate.

**Table 1:** Sequences of primers used in Real time PCR

Primer	Forward (5′ → 3′)	Reverse(5′ → 3′)	Size(bps)
SIRT1	*GCCTTTCTGACCCTAATG*	*CCTCAACAGGAGACAATG*	159
AKT1	*CACACGCTTACTGAGAAC*	*GTTGGCATACTCCATGAC*	114
MuRF1	*TGGGTTTGGAGACAAAGAC*	*AGCGTTTCCATCAGGAATC*	102
GAPDH	*ATCACTGCCACCCAGAAG*	*TCCACGACGGACACATTG*	191

### Western blotting analysis

After lysing the cells with RIPA buffer, the total protein concentration was measured using a BCA protein kit. A 40-µg protein sample was loaded onto a sodium dodecyl sulphate–polyacrylamide gel for electrophoresis, transferred to a polyvinylidene fluoride membrane, and blocked with 5% skim milk containing 0.1% Tween 20 (TBST). Primary antibodies (AKT1, EGFR and SIRT1) were applied at 4℃. The membrane was washed with TBST and incubated with a HRP-labelled secondary antibody for 1 h at room temperature. Protein bands were visualised using a western blot gel analyser, and ImageJ software was used for grey-level analysis (Seo et al. 2022).

### Statistical analysis

SPSS software (v26.0, accessed on 3 July 2025) was used to analyse the experimental data, expressed as mean ± standard deviation. One-way analysis of variance was used to compare group averages, followed by Tukey’s test. A P-value < 0.05 indicated statistical significance (Chang and Kong 2020). Three biological replicates were used in each experiment.

## Results

### Collection of action targets

**Fig. 1:**
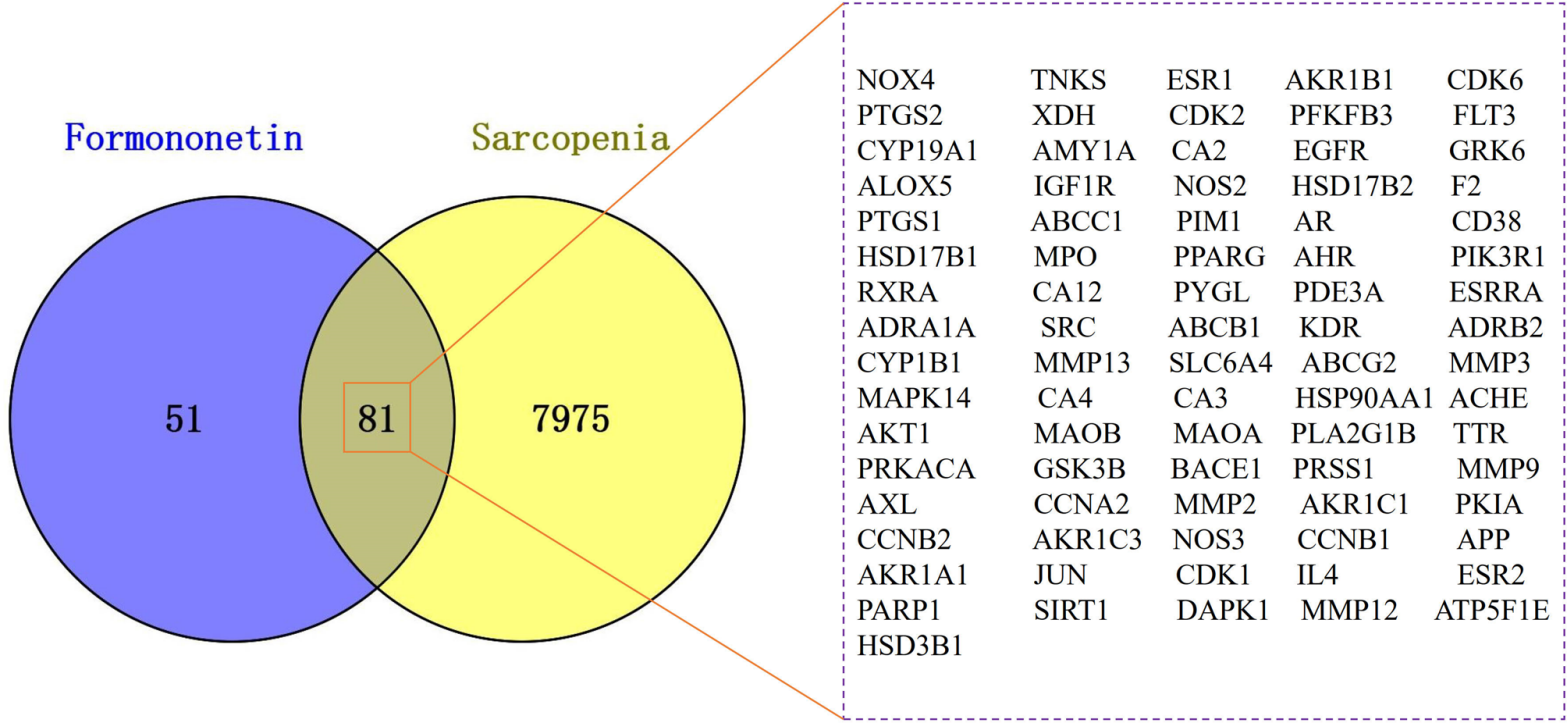
Venny diagram of intersection targets of sarcopenia and sarcopenia. Details of the 81 intersecting targets are in the right box

**Fig. 2:**
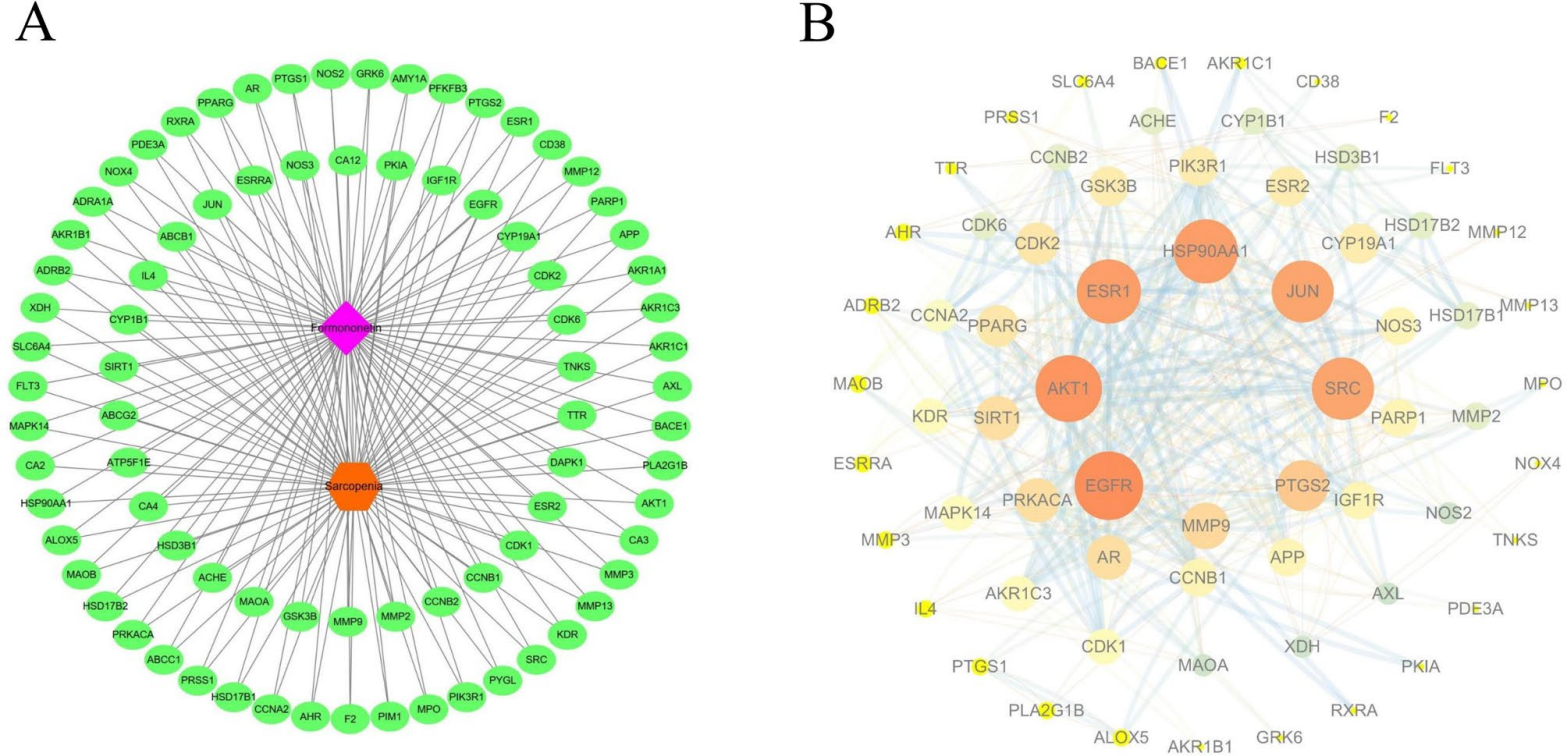
The therapeutic network of FMN. Component-target-disease action network (**A**). PPI network of FMN for treatment of SP (**B**)

After removing duplicates from TCMSP and STP, 132 drug targets were identified. The GeneCards, OMIM, and CTD databases yielded 8,056 SP-related targets after deleting duplicate entries. Mapping the 132 FMN targets to the SP set revealed 81 potential FMN targets for SP treatment (Fig. 1), thereby indicating the ability of FMN to regulate various SP-related pathways. Cytoscape 3.7.2 was used to create a drug-target-disease network diagram. This network, with 191 nodes and 1141 edges, highlighted the relationships between FMN, cross targets, and SP (Chang et al. 2025). The nodes represent compounds, targets, and diseases, whereas the edges represent their interactions (Fig. 2A).

### Identification of core targets

**Fig. 3:**
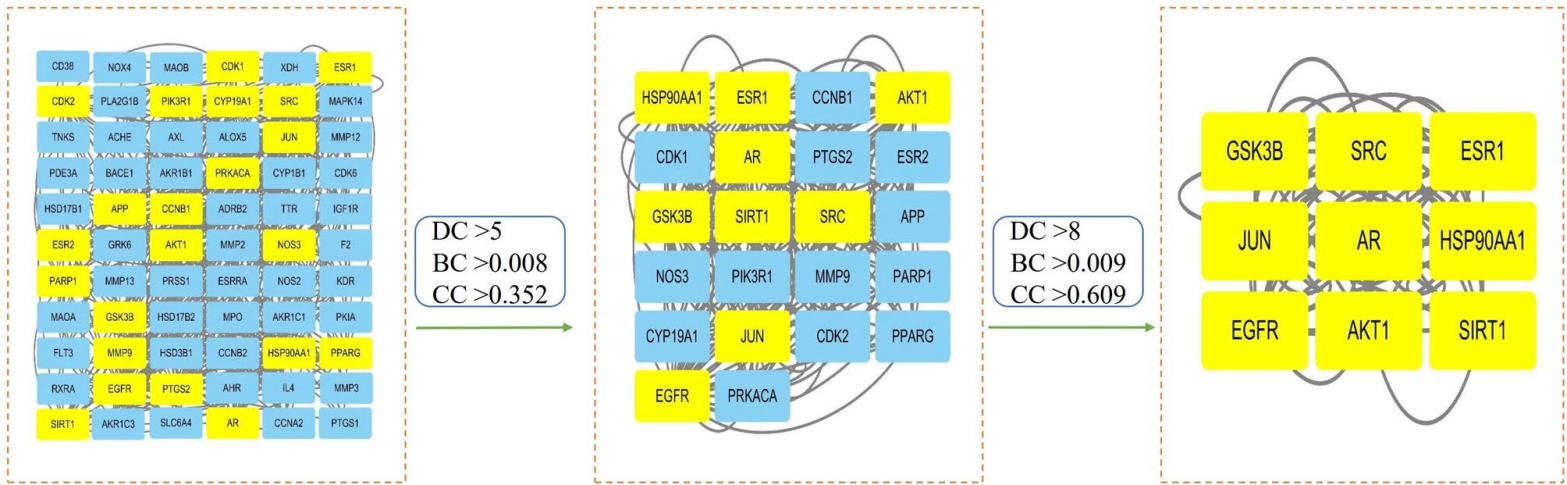
Topological analysis of core target networks

The target proteins were analysed using the STRING database for *Homo sapiens*. This resulted in a refined protein interaction network with 66 nodes and 416 edges after removing target proteins unrelated to the interaction (Fig. 2B). Using the CytoNCA topological analysis in Cytoscape, the targets were first filtered to identify 22 core targets with DC > 5, BC > 0.008, and CC > 0.352. These were further refined to nine core targets with DC > 8, BC > 0.009, and CC > 0.609 (Fig. 3, Table 2).

**Table 2:** The core goal of formononetin against sarcopenia

No	Target	Degree centrality	Between centrality	Closeness centrality
1	JUN	17	0.108	0.840
2	AKT1	16	0.109	0.808
3	ESR1	16	0.094	0.808
4	EGFR	16	0.115	0.808
5	HSP90AA1	14	0.065	0.750
6	SRC	13	0.044	0.724
7	AR	10	0.178	0.656
8	SIRT1	9	0.014	0.636

### Enrichment analysis of GO and KEGG pathways

**Fig. 4:**
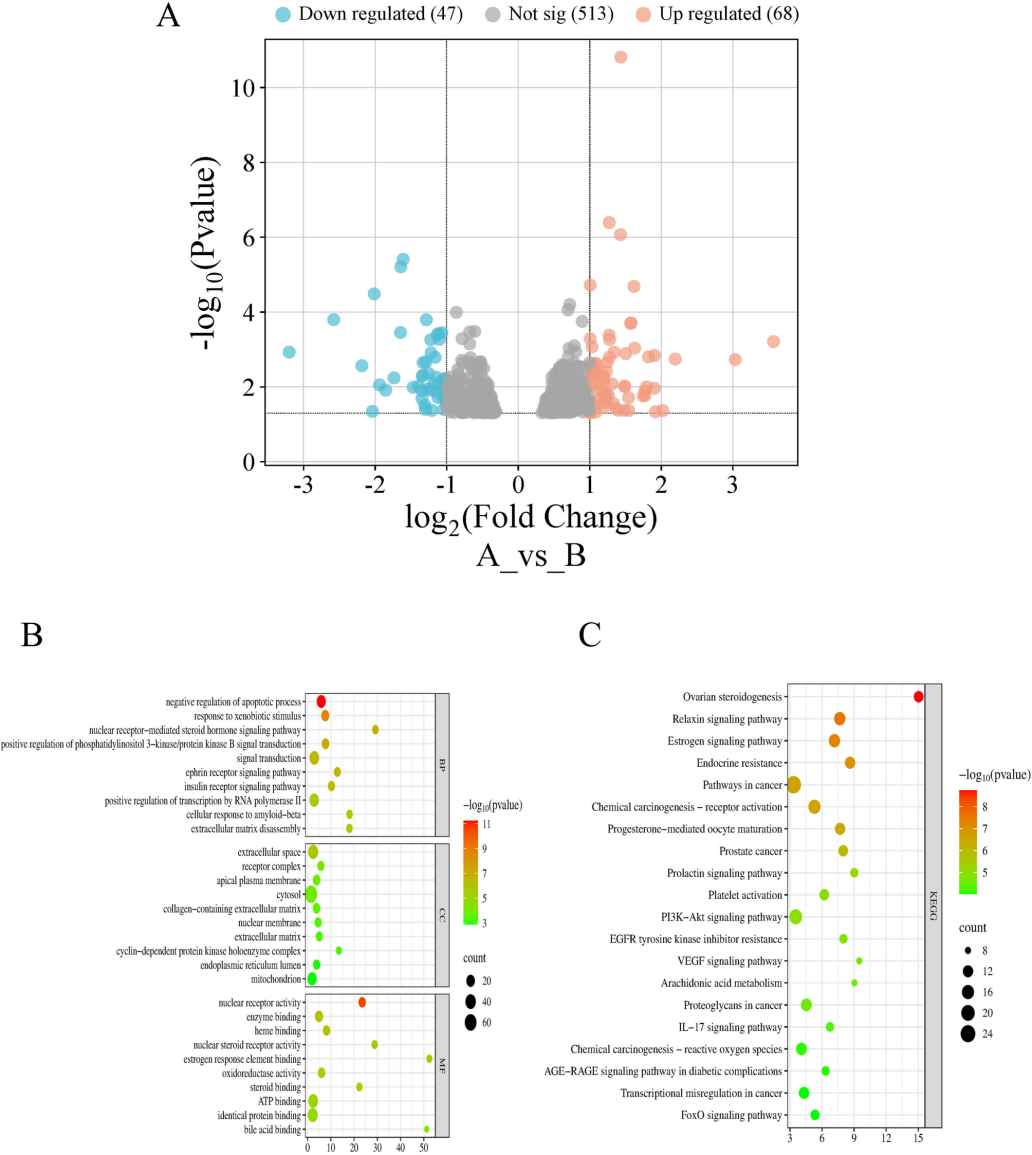
Enrichment analysis of target genes in the treatment of SP with FMN. GEO differentially expressed genes (**A**); GO analysis (**B**); KEGG analysis (**C**)

Using the GSE238215 database with the criteria of |log2FC|> 1 and *p* < 0.05, 115 differentially expressed genes were identified between patients with SP and healthy individuals, with 68 up-regulated and 47 downregulated genes (Fig. 4A). These genes were combined with 81 intersection targets, and GO and KEGG enrichment analyses were conducted using the DAVID database (Zhao et al. 2022). A total of 489 GO terms were identified, including 281 biological processes, 49 cellular components, and 159 molecular functions. The top 10 items with the smallest P-values are shown in Fig. 4B. The biological process terms primarily included negative regulation of the apoptotic process, response to xenobiotic stimuli, response to xenobiotic stimuli, and positive regulation of phosphatidylinositol 3-kinase/protein kinase B signal transduction. The gene products were regulated by the extracellular space, nuclear membrane, extracellular matrix, and mitochondria. The identified molecular functions included enzyme binding, oxidoreductase activity, and adenosine triphosphate binding. KEGG enrichment analysis was used to assess the macro-level impact of FMN on SP by examining key genes (Wu et al. 2023). The top 20 pathways ranked by P-values are shown in Fig. 4C and were primarily enriched in pathways, such as cancer, endocrine resistance, chemical carcinogenesis-reactive oxygen species, phosphatidylinositol 3-kinase (PI3K)-protein kinase B (Akt), IL-17, vascular endothelial growth factor, and forkhead box O (FoxO) signalling.

### MD results

**Fig. 5:**
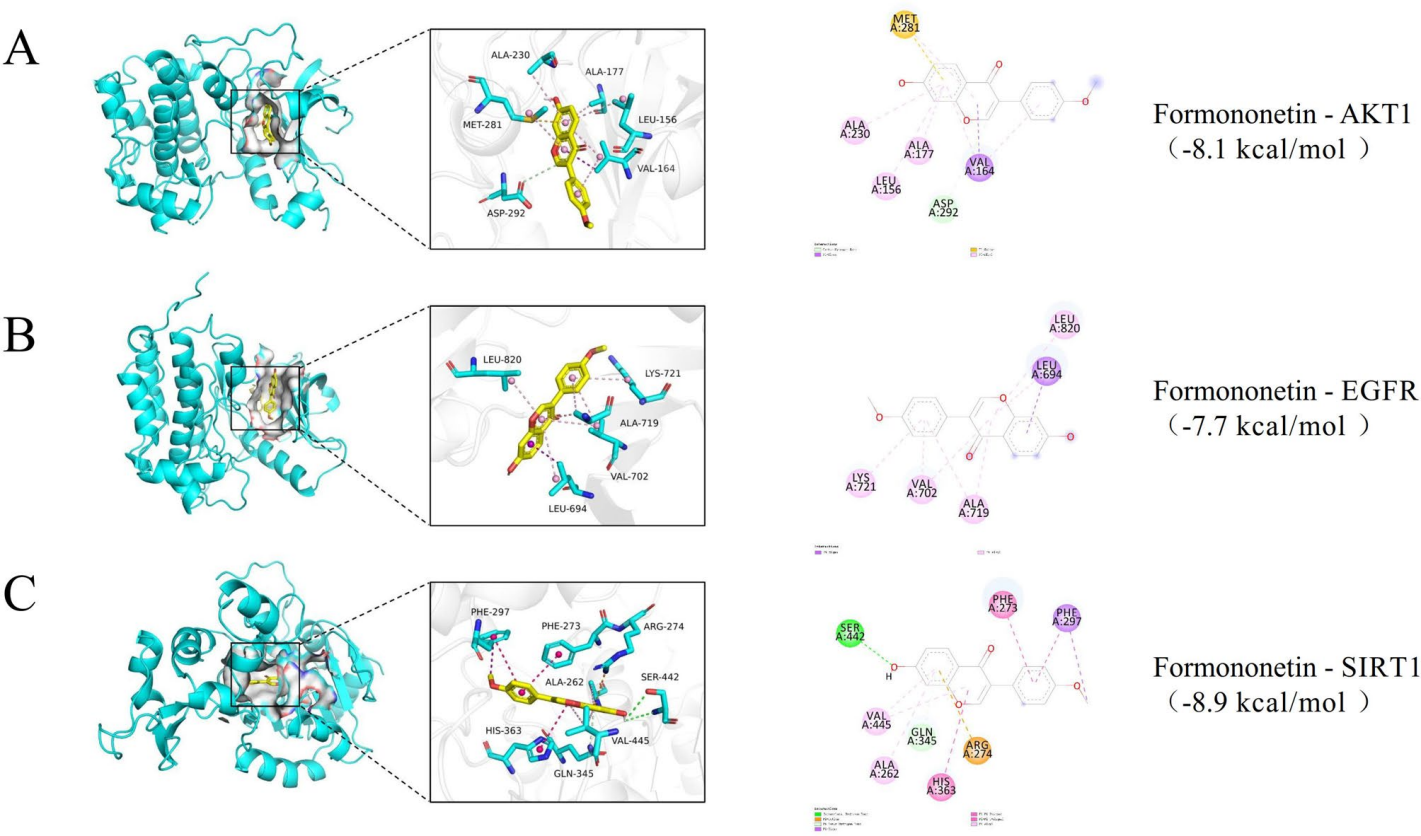
Molecular docking of three core targets with formononetin. Docking of AKT1 and formononetin (**A**); Docking of EGFR and formononetin (**B**); Docking of SIRT1 and formononetin (**C**)

MD was used to analyse the binding activity of FMN to three FoxO signalling pathway targets, (AKT1, EGFR, and SIRT1). A docking score below 0 suggests binding, whereas a score below − 5 kcal/mol indicates strong binding (Xiao et al. 2023). FMN bound well to all the three protein targets with reliable interactions. FMN interacted with AKT1 through carbon hydrogen bonds, pi-sigma, pi-sulfur, and pi-alkyl (Table 3, Fig. 5A). However, the binding of FMN to EGFR was facilitated solely by pi-sigma and pi-alkyl (Table 3 and Fig. 5B). Furthermore, FMN exhibited a diverse range of bonding interactions with SIRT1, including conventional hydrogen bonds, pi-cations, pi-donor hydrogen bonds, pi-sigma, pi-pi stacked, pi-pi T-shaped, and pi-alkyl (Table 3, Fig. 5C). The AutoDock Vina docking parameters are listed in Table 4.

**Table 3:** Sites and forms of formononetin docking with three core targets

Connection method	Connection location of AKT1	Connection location of EGFR	Connection location of SIRT1
Carbon hydrogen bond	ASP (A:292)	–	–
Pi-Sulfur	MET(A:281)	–	–
Conventional hydrogen Bond	–	–	SER(A:442)
Pi-cation	–	–	ARG (A:274)
Pi-donor hydrogen bond	–	–	GLN (A:345)
Pi-sigma	VAL(A:164)	LEU(A:694)	PHE (A:297)
Pi-Pi stacked	–	–	PHE (A:273)
Pi-Pi T-shaped	–	–	HIS (A:363)
Pi-alkyl	ALA (A:230); ALA (A:177); LEU(A156)	VAL (A:702); ALA (A:719); LYS (A:721); LEU (A:820)	VAL(A:445); ALA(A:262)

**Table 4:** AutoDockVina docking parameters of FMN and three core targets

Docking parameter	AKT1	EGFR	SIRT1
center_x	6	22.01	42.96
center_y	3.01	0.25	− 21.41
center_z	17.34	52.79	18.53
size_x	22.5	22.5	22.5
size_y	22.5	22.5	22.5
size_z	22.5	22.5	22.5
exhaustiveness	32	32	32
num_modes	20	20	20
energy_range	3	3	3

### MDS analysis

**Fig. 6:**
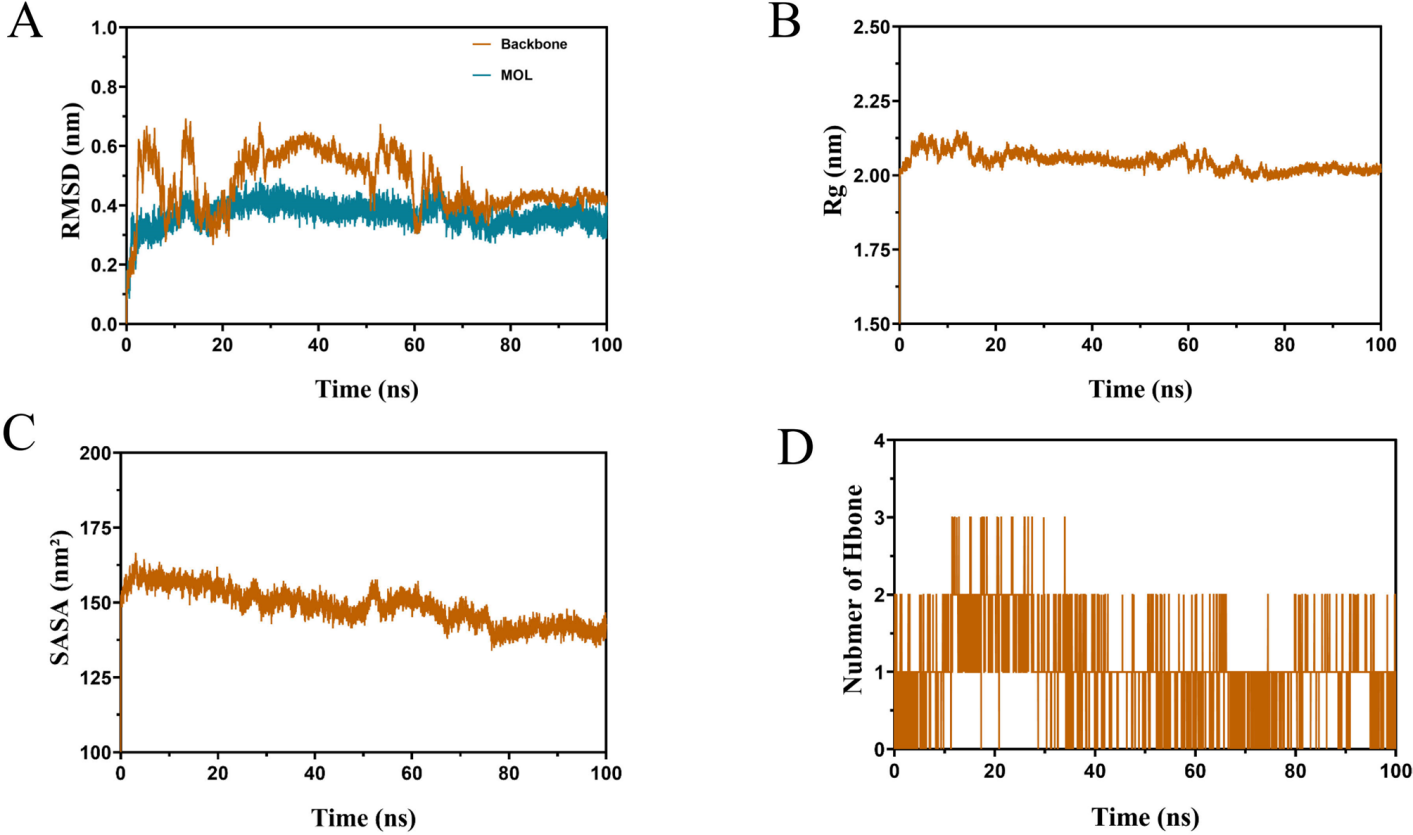
MDS of FMN/SIRT1 complex. Root mean square deviation (RMSD/nm) (**A**); Radius of gyration (Rg/nm) (**B**); Solvent accessible surface area (SASA/nm^2^) (**C**); Hydrogen bonds (**D**)

Docking analyses revealed that FMN exhibited strong binding affinity for SIRT1. MDS corroborated this interaction, with the root-mean-square deviation (RMSD) indicating stabilisation of the FMN/SIRT1 complex after approximately 70 ns (Fig. 6A). The radius of gyration (Rg) demonstrated minor fluctuations around 2.10 nm for the complex, with no significant structural alterations (Fig. 6B). The solvent-accessible surface area (SASA), which reflects the extent of molecular exposure to the solvent and influences the dissolution rate, remained stable for the FMN/SIRT1 complex. The SASA fluctuated between 135 and 165 nm^2^, indicating minimal changes in solvent exposure and a tendency towards stabilisation (Fig. 6C). This was consistent with the RMSD. Hydrogen bonds, which are essential for maintaining interactions between SIRT1 proteins and FMN, averaged around two throughout the simulation period (0–100 ns), with only minor fluctuations. This overall stability underscores the critical role of hydrogen bonds in preserving the structure and function of the complexes (Fig. 6D).

Subsequently, the free energy of the last 20 ns of the system was calculated using the molecular mechanics Poisson-Boltzmann surface area (MM-PBSA) method to understand the protein-small-molecule interactions. Van der Waals forces were the most substantial (− 165.291 ± 0.853 kJ/mol), followed by electrostatic interactions (− 21.957 ± 0.545 kJ/mol) and nonpolar solvation energy (− 15.835 ± 0.069 kJ/mol). Although solvation energy (110.661 ± 0.694 kJ/mol) impeded binding, the strong van der Waals force overcame this, resulting in an interaction energy of − 92.366 ± 0.972 kJ/mol between the protein and ligand.

### ADMET prediction results

The ADMET characteristics of MN were evaluated using SwissADME and pkCSM (Table 5). FMN exhibited a high predicted gastrointestinal absorption. Moreover, FMN was not a substrate for P-glycoprotein and did not inhibit the hepatic enzymes cytochrome P450 (CYP)2C19 and CYP2C9. However, FMN inhibits CYP1A2, CYP2D6, and CYP3A4. In terms of drug-likeness, FMN complied with Lipinski’s rule of five and was considered favourable according to the criteria set by Ghose, Veber, Egan, and Muegge, without inducing adverse effects. Toxicity predictions suggested that FMN was not associated with AMES toxicity, hepatotoxicity, or skin sensitisation, and did not inhibit the human ether-a-go-go-related gene (hERG) channel. In humans, the maximum tolerated dose of FMN is 0.008 mg/kg/day.

**Table 5:** Pharmacokinetics and toxicity characteristic of formononetin

Item	FMN	Item	FMN
GI absorption	High	Egan	Yes
BBB permeant	0.157	Muegge	Yes
Pgp substrate	No	Bioavailability score	0.55
CYP1A2 inhibitor	Yes	Pain	0
CYP2C19 inhibitor	No	Synthetic accessibility	2.81
CYP2C9 inhibitor	No	AMES toxicity	No
CYP2D6 inhibitor	Yes	Max.tolerated dose (human)	0.008
CYP3A4 inhibitor	Yes	hERG I inhibitor	No
Lipinski	Yes	hERG II inhibitor	No
Ghose	Yes	Oral rat acute toxicity (LD50)	1.946
Veber	Yes	Oral rat chronic toxicity (LOAEL)	1.17
Hepatotoxicity	No	T.Pyriformis toxicity	0.637
Skin sensitisation	No	Minnow toxicity	0.041

### Determining optimal concentrations of DEX and FMN for treating C2C12 cells

**Fig. 7:**
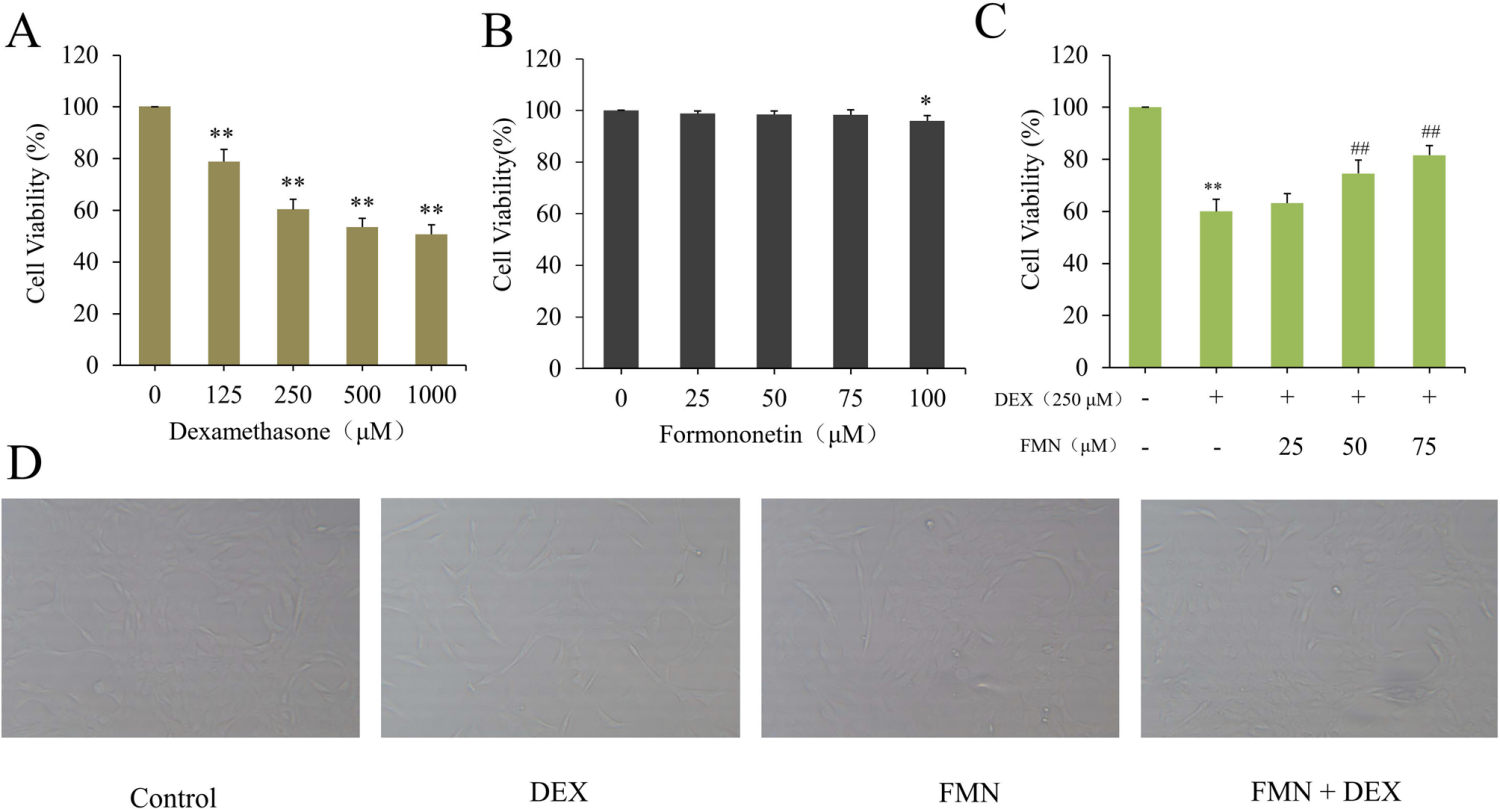
Effect of DEX treatment on the viability of C2C12 cells (**A**); Effect of FMN treatment on the viability of C2C12 cells (**B**); Effect of DEX and FMN treatment on the viability of C2C12 cells (**C**); Morphological changes of C2C12 cells treated with or without DEX or FMN (× 200) (**D**). In Fig. A-C, * *p* < 0.05, * * *p* < 0.01 compared to Control group (0 μM); In Fig. C, ## *p* < 0.01, compared to Model group (DEX); (*‾X* ± *S, n* = *6*)

C2C12 myotube cell viability was assessed after exposure to various DEX concentrations (0, 125, 250, 500, 1000 μM) for 4 h and FMN (0, 25, 50, 75, 100 μM) for 24 h. The results indicated that DEX significantly reduced C2C12 myotube cell viability in a dose-dependent manner (*p* < 0.01) (Fig. 7A), while FMN had no significant impact at or below the test concentration of 75 μM, and a treatment concentration of 100 μM FMN resulted in a significant difference (*p* < 0.05) (Fig. 7B). However, FMN notably enhanced the viability of DEX-treated cells in a dose-dependent manner. Therefore, subsequent tests were performed with 250 μM DEX and 0-75 μM FMN to ensure cell viability remained stable.

### FMN alleviates DEX-induced myotube atrophy in C2C12 cells

Our study demonstrated that FMN enhanced the viability of C2C12 myotube cells treated with 250 µM DEX in a dose-dependent manner (p < 0.01) (Fig. 7C). Additionally, morphological analysis further revealed that 250 µM DEX induced cell atrophy, whereas 75 μM FMN effectively reversed this effect and prevented myotube thinning (Fig. 7D). These findings suggest that FMN plays a key role in inhibiting the atrophy of C2C12 cells.

### Effect of FMN on IL-6 levels and SOD activity in C2C12 cells

**Fig. 8:**
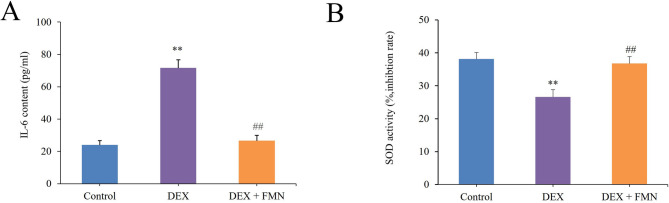
Effects of FMN treatment on IL-6 content (**A**) and SOD activity (**B**) of C2C12 cells induced by DEX. * * *p* < 0.01 vs. Con group; ## *p* < 0.01 vs. Mod group. (*‾X* ± *S, n* = *6*)

As illustrated in Fig. 8A and B, IL-6 levels in C2C12 myotube cells of the model group were significantly elevated compared to those in the control group (*p* < 0.01), whereas SOD activity was significantly reduced (*p* < 0.01). In contrast, the FMN group exhibited a significant decrease in IL-6 levels in C2C12 myotube cells compared with those in the model group (*p* < 0.01). This was accompanied by a significant increase in SOD activity (*p* < 0.01) (Zhang et al. 2013).

### Effect of FMN on DEX-induced gene expression in C2C12 cells

**Fig. 9:**
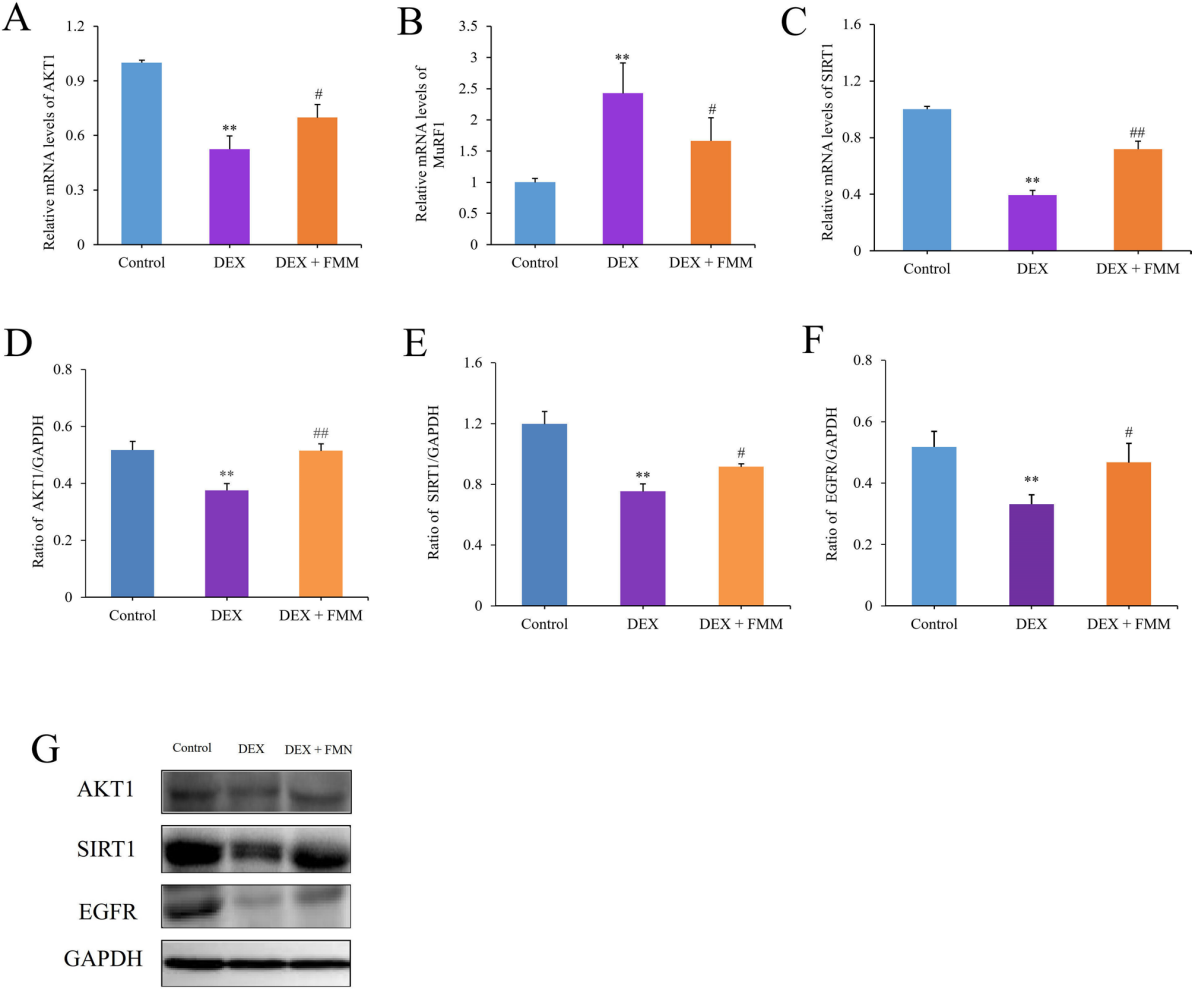
Effects of FNN treatment on the expression of AKT1, MuRF1, and SIRT1genes (**A**–**C**) and AKT1, SIRT1 and EGFR proteins (**D**–**F**) in C2C12 cells induced by DEX. (**G**) Gray strip diagram of three proteins. * * *p* < 0.01 vs. Con group; #*P* < 0.05, ## *p* < 0.01 vs. Mod group. (*‾X* ± *S, n* = *3*)

Real-time PCR was used to quantify the expression of genes related to muscle maintenance in C2C12 myotubes. Figure 9A–C illustrates the effect of DEX induction and FMN intervention on the expression of genes associated with muscle mass and function (i.e. SIRT1, AKT1, and muscle-specific RING finger protein-1 [MuRF1]). DEX induced the expression of MuRF1 in C2C12 myotubes and simultaneously reduced the expression of SIRT1 and AKT1 (*p* < 0.01). These findings suggested that DEX promotes inflammation, disrupts the balance of the antioxidant system, and accelerates muscle cell degradation. Conversely, the addition of FMN to C2C12 myotubes resulted in significant suppression of MuRF1 gene expression (*p* < 0.05), along with a marked increase in SIRT1 and AKT1 gene expression (*p* < 0.05 or *p* < 0.01). This indicated that FMN mitigated the inflammatory response, inhibited muscle cell degradation, and restored the antioxidant system.

### Effects of FMN on SIRT1, EGFR, and AKT1 protein expression in C2C12 cells

Figure 9D–G shows that AKT1, SIRT1, and EGFR expression in model group C2C12 myotubes was significantly lower than that in the control group, at 0.73-, 0.63, and 0.64 times the control levels, respectively (*p* < 0.01). Conversely, in the FMN group, their expression increased significantly compared with those in the model group, reaching 0.99-, 0.77, and 0.90 times the control levels (vehicle-only) (*p* < 0.05 or *p* < 0.01). This suggests that FMN activates AKT by regulating SIRT1 and EGFR levels to relieve SP.

## Discussion

SP is a prevalent aging-associated disease that leads to the loss of muscle mass and strength, thereby creating a need for safe and effective treatments (Lou et al. 2020). Older adults have a considerably lower myoblast determination protein (MYOD) and paired box protein 7 (PAX7)gene expression in muscles than that in younger individuals, thereby suggesting altered gene regulation in muscle stem cells and reduced muscle regeneration (Lai et al. 2024). Thus, inhibiting muscle degradation is a viable approach for treating SP. FMN is effective in countering chronic kidney disease-induced muscle atrophy by modulating the PI3K/Akt/FoxO3a pathway and satellite cell function (Liu et al. 2021). However, its impact on muscle cell morphology and related upstream and downstream protein expression in the aforementioned pathways requires further investigation.

Figure 1 identifies 81 potential targets, including AKT1, EGFR, and Jun proto-oncogene (JUN), for FMN to treat SP. Figure 2A intuitively reflects the complex interactions among components, targets, and diseases, whereas Fig. 2B highlights the target importance using colour and size variations. Iron death, which is a novel form of cell death linked to iron build-up and lipid peroxidation (Zhang et al. 2023), involves the JUN gene (Ma et al. 2024) and impedes chondrocyte growth and accelerates aging (Xie et al. 2023). AKT1 is crucial for initiating myoblast differentiation during muscle regeneration and muscle fibre development (Gardner et al. 2012). The insulin-like growth factor 1 (IGF-1)/PI3K/Akt and Akt/mammalian target of rapamycin (mTOR)/FoxO1 pathways inhibit FoxO transcription factors, thereby preventing the expression of ubiquitin ligases that induce muscle atrophy (Stitt et al. 2004; Wang et al. 2014). In addition, androgens influence skeletal muscle quality. EGFR treatment of C2C12 cells enhances AKT phosphorylation and protein synthesis, thereby suggesting its role in improving muscle quality (Onishi et al. 2025). SIRT1 is a metabolic regulator found in various tissues that combats metabolism and age-related diseases (Chalkiadaki et al. 2014). SIRT1 activation in muscle tissues enhances muscle function by providing anti-inflammatory and antioxidant benefits (Domi et al. 2021). SIRT1 overexpression mitigates muscle loss by inhibiting FoxO1 and FoxO3 activation, thereby preventing muscle-specific ubiquitin ligase and MuRF1 induction, and reducing overall protein breakdown (Lee and Goldberg 2013). Through a comprehensive analysis of the BC, CC, and DC parameters using the CytoNCA plug-in (Fig. 3), AKT1, EGFR, and SIRT1 were determined to be the main targets for FMN to treat SP, which is consistent with the aforementioned analysis. In terms of the interaction between target proteins and compound molecules, the MD results (Fig. 5) corroborate the stable binding of FMN to the three core targets. Moreover, based on the MDS results evidenced by the RMSD, Rg, SASA, and other indicators (Fig. 6), FMN and SIRT1 exhibit stable and strong binding affinities. These findings not only validate the predictive reliability but also elucidate the potential mechanism by which FMN influences SP, thereby offering valuable insights for future experimental investigations.

Based on Fig. 4, the PI3K-Akt, IL-17, and FoxO signalling pathways are integral to SP pathogenesis. IL-17 is a pleiotropic inflammatory cytokine linked to immunity and inflammation. The IL-17 signalling pathway shows increased expression in inflammatory skeletal muscle and is accompanied by higher levels of pro-inflammatory cytokines, such as IL-1β, IL-6, and tumour necrosis factor (TNF)-α (Ge et al. 2023; Liu et al. 2024a, b, c). This suggests its potential for reducing muscle inflammation. Moreover, modulation of the SIRT1-AKT-nuclear factor erythroid 2-related factor 2 (Nrf2) and SIRT1-FoXO3a signalling pathways can mitigate muscle injury. This primarily occurs through mechanisms involving anti-inflammatory effects, reduction of oxidative damage, and inhibition of mitochondria-mediated apoptosis (Zhao et al. 2021). The in vitro results closely aligned with the aforementioned analysis. As depicted in Fig. 8, FMN mitigated inflammatory damage in C2C12 myocytes by decreasing IL-6 levels and further attenuated oxidative damage by enhancing SOD activity.

Excessive glucocorticoid levels contribute to muscle atrophy by promoting protein breakdown via the myostatin pathway (Lee et al. 2022; Wang et al. 2016). DEX likely triggers muscle atrophy by activating cellular protease systems and reducing IGF-1 production, which, in turn, decreases muscle synthesis (Schakman et al. 2009; Girón et al. 2015). In this study, DEX concentrations of 125 μM or higher severely damaged C2C12 muscle cells (Fig. 7A, D). Inflammation and oxidative stress are crucial for muscle regeneration, with IL-6 and SOD being the key players (Kim et al. 2023). In this study, 75 μM FMN intervention effectively mitigated these trends (Fig. 7B, D), thereby restoring balance to inflammation and oxidative stress (Fig. 8). Additionally, FMN treatment boosts AKT1 and SIRT1 mRNA and protein levels while inhibiting MuRF1 mRNA expression and increasing EGFR protein expression (Fig. 9). Activation of EGFR leads to the activation of PI3K, which activates the PI3K/AKT/mTOR signalling pathway and promotes the differentiation of C2C12 cells (Teng et al. 2025; Yang et al. 2016). These findings underscore the critical regulatory roles of SIRT1 and EGFR. These targets may considerably attenuate muscle inflammation and enhance antioxidant levels, thereby mitigating muscle degradation through activation of the PI3K/AKT/mTOR signalling pathway.

Many compounds used for SP treatment share similar effects and mechanisms with those of FMN. Quercetin, a natural polyphenol flavone, has anti-inflammatory and antioxidant properties (Le et al. 2014). As a dietary supplement, it inhibits TNF-α-induced MuRF1 expression, thereby preventing muscle atrophy (Kim et al. 2018). Quercetin glycosides can counteract DEX-induced gastrocnemius muscle weight loss, reduce muscle atrophy gene expression, and increase Akt phosphorylation (Otsuka et al. 2019). Similarly, resveratrol serves as an activator of the NAD^+^-dependent protein deacetylase SIRT1, which inhibits SP by restoring autophagy in mice (Hori et al. 2011). Furthermore, resveratrol administration at a dose of 150 mg/kg/day markedly enhanced relative grip strength and muscle mass in aged rats. Resveratrol also increases sarcomere length, as well as the dimensions of the I-band and H-band. This is potentially because of its protective effects on skeletal muscle through the activation of the AMPK/SIRT1 anti-apoptotic signalling pathway (Liao et al. 2017). Long-term supplementation with 20 mg/kg resveratrol can combat SP in naturally aging C57BL/6 mice by markedly reducing cyclooxygenase-2 levels in muscle tissue through anti-inflammatory effects (Sirago et al. 2022). In addition, β-hydroxy-β-methylbutyrate (HMB) effectively mitigates the enhancement of protein degradation and the suppression of DEX-induced protein synthesis. This is achieved through the regulation of cell signalling pathways dependent on p38/ mitogen-activated protein kinase and PI3K/Akt, thereby attenuating myotube atrophy (Chen et al. 2025; Liu et al. 2024a, b, c). Given the early and gradual onset of SP, the timing of interventions may be crucial for achieving beneficial outcomes. Considering that FMN exhibits a comparable effect in mitigating myotube atrophy and modulating the SIRT1/Akt pathway, akin to the aforementioned compounds, and considering prior ADMET predictions, FMN holds promising potential for application in the treatment of SP.

## Conclusions

This study investigated the molecular mechanisms of FMN in the treatment of SP using NP prediction and experimentation. These findings suggest that FMN has the potential to enhance myotube cell differentiation and reduce muscle degradation by activating AKT1 and SIRT1, while simultaneously inhibiting MuRF1 expression. This supports its potential use for SP treatment. Considering that FMN is widely found in medicinal and edible plants, the compound has the potential to be incorporated into daily or clinical dietary supplements. This flavonoid compound, which has anti-inflammatory and antioxidant effects, has good oral bioavailability (Luo et al. 2018) and may play an alleviating role in a variety of conditions, such as neurodegenerative diseases (Sugimoto et al. 2021) and fractures (Huang et al. 2018). Further in vivo studies and clinical research are needed to enhance scientific understanding of these interventions.

## Data Availability

All data generated or analyzed during this study are available from the corresponding author on reason able request.

## Abbreviations

SP: Sarcopenia
FMN: Formononetin
IL: Interleukin
EGFR: Epidermal growth factor receptor
SIRT1: Sirtuin 1
NP: Network pharmacology
TCMSP: Traditional Chinese Medicine Systems Pharmacology
SMILES: Simplified Molecular Input Line Entry System
CTD: Comparative Toxicogenomics Database
OMIM: Online Mendelian Inheritance in Man
PPI: Protein–protein interaction
STRING: Search Tool for the Retrieval of Interacting Genes/Proteins
DC: Degree centrality
CC: Closeness centrality
BC: Between centrality
GO,: Gene ontology
KEGG: Kyoto Encyclopedia of Genes and Genomes
DAVID: Database for Annotation, Visualization, and Integrated Discovery
MD: Molecular docking
MDS: Molecular dynamics simulations
ADMET: Absorption, distribution, metabolism, excretion, and toxicity
FBS: Foetal bovine serum
DMSO: Dimethyl sulfoxide
RIPA: Radioimmunoprecipitation assay
DEX: Dexamethasone
CCK-8: Cell Counting Kit-8
PCR: Polymerase chain reaction
DMEM: Dulbecco’s Modified Eagle’s Medium
BCA: Bicinchoninic acid
SOD: Superoxide dismutase
TBST: 0.1% Tween 20
PI3K: Phosphatidylinositol 3-kinase
Akt: Protein kinase B
FoxO: Forkhead box O
RMSD: Root-mean-square deviation
Rg: Radius of gyration
SASA: Solvent-accessible surface area
CYP: Cytochrome P450
MuRF1: Muscle-specific RING finger protein-1
Jun: Jun proto-oncogene
IGF-1: Insulin-like growth factor 1
mTOR: Mammalian target of rapamycin
TNF: Tumour necrosis factor
